# Detection of Methicillin-Resistant *Staphylococcus aureus* Infections Using Molecular Methods

**DOI:** 10.3390/antibiotics11020239

**Published:** 2022-02-12

**Authors:** Fred C. Tenover, Isabella A. Tickler

**Affiliations:** Cepheid, Sunnyvale, CA 94089, USA; isabella.tickler@cepheid.com

**Keywords:** MRSA, MSSA, PCR, *mecA*, blood culture, methicillin resistance

## Abstract

The application of molecular detection methods for bacterial pathogens has dramatically improved the outcomes of septic patients, including those with methicillin-resistant *Staphylococcus aureus* (MRSA) infections. Molecular methods can be applied to a variety of clinical specimens including nasal swabs, growth in blood culture bottles, and wounds. While data show that the overall accuracy of molecular tests for MRSA is high, results can be confounded by the presence of multiple staphylococcal species in a specimen, insertions and deletions of DNA in and around the Staphylococcal Cassette Chromosome *mec* (SCC*mec*) element, and point mutations in *mecA*. Herein, we explore the complexities of molecular approaches to MRSA detection and the instances where phenotypic methods should be pursued to resolve discrepancies between genotypic and phenotypic results.

## 1. Introduction

The application of molecular detection methods for bacterial pathogens has significantly improved the outcomes of septic patients, especially in conjunction with antimicrobial stewardship programs [1]. This includes patients with infections caused by *Staphylococcus aureus* and particularly those with methicillin-resistant *S. aureus* (MRSA) [2,3]. MRSA infections continue to be a significant cause of morbidity and mortality worldwide and still can be a challenge to treat effectively [4,5]. 

The discovery of the *mecA* gene as the key mechanism of methicillin resistance in staphylococcal species enabled the development of rapid molecular tests to distinguish between colonies of MRSA and colonies of methicillin-susceptible *S. aureus* (MSSA), first by DNA probes and then by PCR [6,7]. However, direct detection of MRSA in clinical specimens was more of a challenge, since *mecA* may also be present in methicillin-resistant strains of coagulase-negative staphylococci, which can also be found in a variety of clinical specimens [8,9,10]. The elucidation of the structure of the Staphylococcal Cassette Chromosome *mec* (SCC*mec*) element, however, enabled a way of linking the *mecA* gene specifically to the chromosome of *S. aureus* [11,12]. In fact, early diagnostic tests for detection of MRSA in nasal specimens relied only on the *orfX-SCCmec* junction region and did not utilize simultaneous detection of *mecA* [13]. This was done by placing one set of primers in the *orfX* locus in the *S. aureus* chromosome and the other in the SCC*mec* element, thus, spanning the SCC*mec* integration site. This paved the way for detection of both MRSA and MSSA directly in a variety of clinical specimens, including wound swabs, organisms growing in blood culture bottles, and samples from osteoarticular infections [14,15,16,17]. Yet, the diversity and complexity of the various SCC*mec* elements that have evolved over time have also proven to be a challenge for molecular tests. Herein, the complexities of molecular approaches to MRSA and MSSA detection will be explored.

## 2. Detection of MRSA Nasal Colonization

The junction region between *orfX* in the *S. aureus* chromosome and the inserted SCC*mec* element was targeted early on for detection of MRSA by PCR directly in nasal swabs [13,18]. While this approach has overall been very successful [13,19,20,21], the loss of the *mecA* gene from the SCC*mec* element (so called “empty cassettes”) resulted in some false positive results in commercial tests [22,23,24]. This was addressed in some tests by adding additional primer sets targeting either *mecA* [25] or both *mecA* and *mecC* to complement those for the junction region to ensure that the methicillin resistance gene was still present in those strains in which the *orfX*/SCC*mec* junction was detected [26,27].

In 2019, the American Thoracic Society and the Infectious Diseases Society of America issued guidelines recommending the use of negative PCR tests targeting MRSA in nasal swabs to guide discontinuation of vancomycin therapy for patients from the United States with community-acquired pneumonia who did not have a history of recent foreign travel and were not immunosuppressed, but who were at risk for MRSA infection [28]. The recommendation to discontinue vancomycin was supported by a meta-analysis of 22 studies and focused on the availability of PCR results in 2 hours versus 2 days by culture. [29]. One additional study validated the cost-effectiveness of this screening approach using a commercial PCR assay [30]. While many institutions have adopted this recommendation, it should be noted that the use of commercial PCR tests for this indication would be considered off-label and would require each institution to perform a validation study before using the test to guide therapeutic strategies. 

## 3. Detecting MRSA and MSSA in Positive Blood Culture Bottles and Skin and Soft Tissue Specimens 

Use of molecular methods to rapidly differentiate MRSA from MSSA in positive blood culture bottles and directly from wound specimens provides a way to optimize treatment for *S. aureus* infections prior to the results of cultures becoming available. Several nucleic acid amplification methods including PCR, microarrays, and isothermal loop-mediated amplification (LAMP) assays have been described [31] as well as matrix-assisted laser desorption ionization time of flight (MALDI-TOF) to rapidly identify colonies isolated from blood culture bottles or directly from positive blood culture bottles prior to obtaining colonies in pure cultures [32]. All of these methods offer reduced time-to-initiation of optimal therapy over traditional culture methods, which require 72 to 96 hours for species identification and antimicrobial susceptibility testing to be completed [33]. Overall, multiple reports show that this strategy of using nucleic acid amplification methods works well and provides accurate results in a timely manner [15,34,35,36,37]. However, there are several genetic variants that have arisen among *S. aureus* strains that can challenge the accuracy of molecular test results. These include variations in SCC*mec* sequences and variations in the chromosomal loci used as targets in the tests to identify organisms as *S. aureus*, such as *spa* and *nuc* [38,39]. For example, similar to tests on nasal swabs, the occurrence of empty cassette strains of *S. aureus*, where the majority of the SCC*mec* element remains in the *S. aureus* chromosome [35,40,41] but the *mecA* gene is excised, can lead to overcalling of MRSA infections with organisms that are phenotypically MSSA. 

A second challenge is the presence of multiple staphylococcal species containing *mecA* in blood culture bottles and wound specimens. The presence of *mecA* in coagulase-negative staphylococci, which are a frequent cause of blood culture contamination, together with MSSA, occasionally led to misdiagnosis of MRSA bacteremia [40,42]. This issue will be discussed in greater detail below. 

A third challenge is point mutations in *mecA* that can result in a phenotypic change from methicillin resistance to susceptibility [43]. Interestingly, such mutations, often in regions of tandem base repeats, may be reversed in the presence of a semi-synthetic penicillin restoring the MRSA phenotype [44]. These strains are often referred to as stealth MRSA or oxacillin-susceptible MRSA (OS-MRSA) [45]. Such MRSA strains have appeared in wound specimens and originally were classified as “oxacillin-inducible” strains, where the phenotype changed from oxacillin-susceptible to resistant after overnight exposure to cefoxitin [46]. These OS-MRSA isolates also changed from PBP2a negative to positive with commercial latex agglutination tests and showed growth on MRSA selective agar media after exposure to cefoxitin. Subculturing a presumptive OS-MRSA strain on a Mueller-Hinton or blood agar plate with a cefoxitin disk in the middle of the growth, and setting up minimal inhibitory concentration (MIC) and disk diffusion tests using growth taken from the edge of the zone of inhibition around cefoxitin disk after 18 to 24 hours of incubation, can confirm the presence of OS-MRSA, as the organism will now be oxacillin resistant [47]. This can resolve some genotype–phenotype discrepancies. 

## 4. Further Exploring the Discrepancies between Genotypic and Phenotypic Results 

The SCC*mec* elements detected among various MRSA strains have proven to be more complex than originally imagined [48]. This has made detection of some novel MRSA strains more difficult with molecular tests [12,49,50,51]. Several examples of MRSA strains that initially went undetected due to insertions of other genetic elements into the *orfX*-SCC*mec* junction region have been reported [40,49]. These included SCC_M1_, ΨSCC6838, and the arginine catabolic metabolic elements (ACME) [52,53], which may integrate through the actions of the CcrA and CcrB functions encoded by the SCC*mec* element [54,55]. These insertions effectively increased the distance between the forward and reverse primer sets directed to *orfX* and SCC*mec,* respectively, by up to 14kb, prohibiting detection by PCR. Some commercial tests have been updated to better differentiate MRSA from MSSA to overcome these issues [40,42]. 

Another source of discrepancies between genotypic results and the phenotypic tests of colonies cultured from specimens is the presence of mixed species of staphylococci, particularly in blood culture bottles [56]. This may be a combination of MRSA and MSSA isolates, MSSA and methicillin-resistant coagulase-negative staphylococci, or variants of the same MRSA strain that undergo deletions or insertions of DNA in vivo [57,58]. The discrepancies can be a result of the large disparity in the concentrations of the two organisms present in the specimen, which can differ by as much as a 1000-fold. Thus, the predominant organism in the specimen often appears to present in pure culture. However, the sensitivity of PCR detects the targets present in both the high and low concentration organisms which, when taken together, may lead to a discrepant result [57]. For example, one commercial test that detects and differentiates between MRSA and MSSA from blood culture bottles does so by targeting *spa*, *mecA,* and the *orfX*-SCC*mec* junction region [40]. When an MSSA strain with an empty SCC*mec* cassette (i.e., one where the *spa* and *orfX*-SCC*mec* targets are positive by PCR but negative for *mecA* due to its excision) is present in a specimen with a methicillin-resistant *Staphylococcus epidermidis,* the molecular test will detect all three targets and report the presence of MRSA. However, the *S. epidermidis* may be in low concentration and not be apparent in the culture, resulting in what appears to be a pure culture of MSSA. Hence, the discrepancy between genotype and phenotype. Culturing an additional aliquot of the specimen on oxacillin-selective agar to aid in the detection of the methicillin-resistant *S. epidermidis* (which harbors *mecA*) is one of several steps that can be taken to resolve this discrepancy [59]. However, one should not assume that every discrepancy between a genotypic report of MRSA and the recovery of an MSSA in culture is due to the presence of coagulase-negative staphylococci. As Tickler et al. have reported, the second organism may well be an MRSA in low concentration and not a coagulase-negative staphylococcus [57]. In this case, it may be the same organism, but the MSSA variant outgrew the MRSA, which can happen due to the fitness cost of *mecA* carriage [60,61], leading to an apparent discrepancy. As the two isolates are, in the example, the same strain, differentiating the colonies on agar plates in the absence of selective pressure is virtually impossible because they will share the same colonial morphology. Use of selective agar plates is imperative to resolve the issue. While a coagulase-negative staphylococcus may be dismissed as a contaminant by clinicians, the presence of an MRSA as the second organism may be viewed differently and may impact the therapeutic choices for the infection. 

Finally, deletions of sequences starting in and around the SCC*mec* element and encompassing *mecA* can extend into the surrounding chromosomal regions and affect other genes that may be used for *S. aureus* identification, such as *spa* and *nuc.* This results in an MSSA phenotype but may also lead to a negative result for *S. aureus* since the target used to identify *S. aureus* is now absent [57,62]. 

## 5. Whole Genome Sequencing as a Diagnostic Tool

WGS has been an important tool for studying the mechanisms of antimicrobial resistance in MRSA [63], including defining novel mechanisms of daptomycin and fusidic acid resistance [64]. WGS has also informed investigations of outbreaks and the changing epidemiology of MRSA strains [65,66] and potentially can aid in predicting the outcomes of staphylococcal infections based on the presence of key virulence determinants [64]. Yet, as a routine diagnostic tool in a hospital laboratory, WGS continues to have several drawbacks. First, there are no WGS-based methods that have received regulatory approval. This is a significant drawback for using WGS for patient care. Second, WGS is more expensive than culture and PCR-based diagnostic tests, especially when the costs of instruments and reagents to perform nucleic acid extraction, library preparation, data assembly, and interpretation are considered [67,68]. Third, the time to final results for WGS is slower than for culture and PCR [69]. While the actual sequencing time gets shorter and shorter, all the steps of testing including pre-analytical, analytical, and post analytical procedures must be considered. Fourth, at present, there are no uniform standards for data interpretation or quality control to insure accurate results [33]. How one translates a genotype, which may be the presence or absence of specific genetic determinants, mutations, and regulatory elements, to a clinical result that will be meaningful and actionable for physicians remains largely to be determined [64]. There are very few studies that address issues such as error rates, reproducibility, and accuracy of WGS methods performed on significant numbers of clinical specimens to guide implementation of this technology. The lack of well-curated databases and data from outcomes studies that support translating genotypes to patient care pathways, beyond those for predicting antimicrobial resistance, remain as important barriers [33,64]. Furthermore, as opposed to standard culture and PCR methods, which have received regulatory approval and are eligible for third-party reimbursement, WGS is not reimbursable, as it is still considered a research method. This, too, is a significant barrier to uptake and utilization by clinical laboratories.

## 6. Conclusions

Molecular diagnostics have dramatically improved the therapy of MRSA and MSSA infections globally. While culture methods remain important due to the need for extended antimicrobial susceptibility testing, PCR-based methods offer more rapid results, which reduces the time to optimal antimicrobial therapy initiation. WGS is a potentially valuable diagnostic tool but costs, slower time to results, lack of approved methods, and the absence of established databases and guidelines for interpreting results are barriers to implementing this technology routinely for patient care. For PCR-based tests, sequence changes in the *S. aureus* chromosome and the relative instability of the SCC*mec* elements pose ongoing challenges to the continued accuracy of the tests. Updated commercial assays have addressed some of the issues. When contradictions between the phenotype of isolated colonies from blood culture bottles or wound specimens and the genotype produced by a molecular method are observed, it is worthwhile to undertake additional selective culture methods, cefoxitin induction, or DNA sequence analysis to resolve the discrepancy.

## Data Availability

Not applicable.
